# Development of an Aptamer-Based Concentration Method for the Detection of *Trypanosoma cruzi* in Blood

**DOI:** 10.1371/journal.pone.0043533

**Published:** 2012-08-22

**Authors:** Rana Nagarkatti, Vaibhav Bist, Sirena Sun, Fernanda Fortes de Araujo, Hira L. Nakhasi, Alain Debrabant

**Affiliations:** Laboratory of Emerging Pathogens, Division of Emerging and Transfusion Transmitted Diseases, Center for Biologics Evaluation and Research, U. S. Food and Drug Administration, Bethesda, Maryland, United States of America; Federal University of São Paulo, Brazil

## Abstract

*Trypanosoma cruzi*, a blood-borne parasite, is the etiological agent of Chagas disease. *T. cruzi* trypomastigotes, the infectious life cycle stage, can be detected in blood of infected individuals using PCR-based methods. However, soon after a natural infection, or during the chronic phase of Chagas disease, the number of parasites in blood may be very low and thus difficult to detect by PCR. To facilitate PCR-based detection methods, a parasite concentration approach was explored. A whole cell SELEX strategy was utilized to develop serum stable RNA aptamers that bind to live *T. cruzi* trypomastigotes. These aptamers bound to the parasite with high affinities (8–25 nM range). The highest affinity aptamer, Apt68, also demonstrated high specificity as it did not interact with the insect stage epimastigotes of *T. cruzi* nor with other related trypanosomatid parasites, *L. donovani* and *T. brucei*, suggesting that the target of Apt68 was expressed only on *T. cruzi* trypomastigotes. Biotinylated Apt68, immobilized on a solid phase, was able to capture live parasites. These captured parasites were visible microscopically, as large motile aggregates, formed when the aptamer coated paramagnetic beads bound to the surface of the trypomastigotes. Additionally, Apt68 was also able to capture and aggregate trypomastigotes from several isolates of the two major genotypes of the parasite. Using a magnet, these parasite-bead aggregates could be purified from parasite-spiked whole blood samples, even at concentrations as low as 5 parasites in 15 ml of whole blood, as detected by a real-time PCR assay. Our results show that aptamers can be used as pathogen specific ligands to capture and facilitate PCR-based detection of *T. cruzi* in blood.

## Introduction

*Trypanosoma cruzi* is the etiological agent of Chagas disease in humans. The disease affects 5–9 million people primarily in Mexico, Central and South American countries and is responsible for more than 500,000 fatalities annually [1]. The parasite is an intracellular pathogen and invades human tissues via wounds or exposed mucus membranes [1]. On infection, some individuals may show disease symptoms within 1–2 months of infection, with parasites detectable in blood, however a majority of them remain asymptomatic. Following this acute phase, infected individuals enter a chronic phase during which an estimated 20–30% of infected individuals will develop clinical symptoms associated with Chagas disease [2], [3], [4].

Current methods of blood donor screening or diagnosis rely primarily on serological tests although these tests could miss individuals with low antibody titers [5]. Currently, there are no commercial, approved PCR assays available for the diagnosis of Chagas disease. However, PCR has been widely used to detect *T. cruzi* in blood by reference laboratories. The extracellular *T. cruzi* trypomastigote forms can be detected in infected individuals by PCR with high sensitivity during the acute phase of the disease when circulating parasite numbers are very high [3]. However, in the time period following parasite inoculation into the host and the beginning of the acute phase of the disease, parasites are difficult to detect in blood by PCR. This is due to the low innoculum levels during a natural infection and the time needed for the parasites to multiply as intracellular amastigotes in tissue. This period is can be defined as the window period for PCR detection. Thus, recently infected individuals may obtain a false negative result by PCR. Similarly, during the chronic phase of disease the number of circulating parasites in blood can fluctuate over time and be difficult to detect by PCR [6], [7]. Under these conditions, if a single parasite is not present in the volume of blood sample used for testing, a negative PCR result will be obtained. Other parameters such as methods used for nucleic acid purification, primers used to amplify parasite DNA and thermo-cycling conditions have resulted in highly variable estimates of sensitivities [5], [7], [8].

An alternative approach for the detection of *T. cruzi* parasites in blood is to utilize highly specific ligands, such as RNA aptamers described in this report, that bind to parasites. These ligands can be incorporated onto paramagnetic beads or filtration devices where parasites in contaminated blood can be captured and assayed using PCR or other detection technologies [9]. This strategy of parasite concentration may facilitate the detection of *T. cruzi* in whole blood by allowing the use of a large volume of sample in these assays. Aptamers are short nucleotide sequences that have been selected to bind specifically and with high affinity to their targets. The targets can be small molecules; proteins, or whole cells [10], [11], [12]. The specificity of aptamer binding is derived from their tertiary structure and by hydrophobic and ionic interactions with the target. Aptamers are developed using *in-vitro* selection protocols comprising of iterative steps where a large library of nucleic acids made of random sequences flanked by specific primer binding sites, is allowed to interact with the target. The bound molecules are purified and amplified using PCR and these steps are repeated iteratively with increasing stringency. This process is known as Systematic Evolution of Ligands by Exponential enrichment (SELEX). To develop RNA aptamers a DNA library is used in an *in-vitro* transcription reaction and RNA produced is utilized for subsequent rounds of target binding and recovery of bound aptamers by reverse transcriptase PCR. At the end of this iterative procedure the sequences that survive the selection pressure are cloned and evaluated for their binding properties, such as, affinity and specificity. Aptamers have been shown to bind with affinities that are in the range of monoclonal antibodies or lower. Their selection can be performed against complex targets such as whole cells, without *a priori* knowledge of a specific target. Base analogues incorporated into the aptamer sequence can also make them stable to serum nucleases [13].

Here we report the development of RNA aptamers that bind with high affinities to live *T. cruzi* trypomastigotes. The specificity and potential use of the aptamers for parasite concentration to facilitate PCR based detection of *T. cruzi* is discussed.

## Results and Discussion

### Generation and Binding Characteristics of *T. cruzi* RNA Aptamers

**Figure 1:**
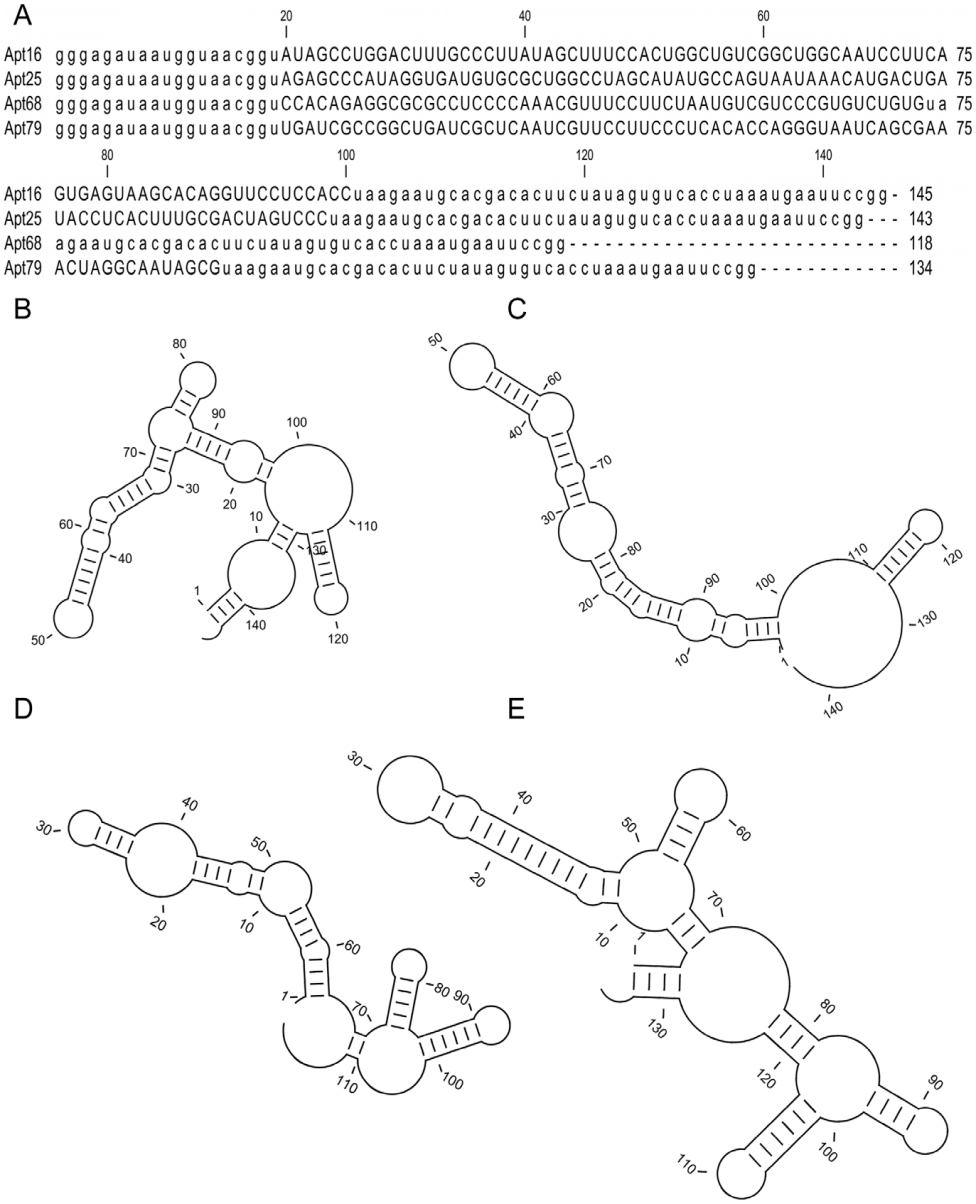
*T. cruzi* whole cell binding aptamer sequences. Nucleotide sequence including the conserved T7 and SP6 primer binding sites, depicted in lower case letters, of the four selected RNA aptamers, Apt16, Apt25, Apt68 and Apt79, obtained from the *T. cruzi* trypomastigote whole-cell SELEX is represented (A). The length of each aptamer sequence is indicated on the right. Predicted secondary structure obtained from Minimal Free Energy (MFE) calculations of Apt16, 25, 68 and 79 are shown in panels B, C, D and E, respectively.

We developed a SELEX strategy to generate RNA aptamers that bind with high affinity and specificity to live *T. cruzi* trypomastigotes. In this SELEX, a pool of short DNA sequences, containing a central 80 nucleotide long randomized region, flanked by conserved T7 and SP6 polymerase binding sites was used in an *in-vitro* transcription reaction to generate a RNA library. Due to the random region, this RNA aptamer library was estimated to contain >10^12^ unique sequences. Nucleotide analogues 2′ F-dUTP and 2′ F-dCTP (fluorinated deoxynucleotides) were incorporated during the transcription reaction to make these RNA molecules resistant to serum nucleases [14]. This RNA library was utilized to select for aptamers that bind to *T. cruzi* trypomastigotes. Refolded aptamer pools were incubated with live trypomastigotes and after several washes the parasite bound aptamers were recovered using RNA extraction procedures. The experimental conditions used during the whole cell SELEX are described in Table S1. The conditions included steps where 5% FBS and salmon sperm DNA were used to decrease non-specific binding of RNA to the parasites as well as negative selection steps where aptamers were bound to blood components or to plastic tubes before binding to trypomastigotes (Table S1). These steps were performed to prevent the selected library from being overrun by sequences that were binding due to non-specific interactions. After 12 rounds of selection, the library was cloned and 100 clones were sequenced. Sequence analysis of these clones indicated a variation in the base pair (bp) length of the random region of the aptamers, resulting in some aptamers with reduced size (ranging from 170 bp to 144 bp). All the aptamers in the starting library had the same length (170 bp). Because of the multiple rounds of PCR amplification and *in-vitro* RNA transcription performed during the SELEX procedure, some sequence deletion may occur due to errors by Taq or T7 polymerase enzymes and this could explain the variability in length of the aptamers recovered [15], [16]. Phylogenetic analysis of the sequences indicated that the library had converged to 4 major families representing >78.3% of the sequenced clones (Figure S1). Individual clones were amplified using T7 and SP6 primers as described in methods section, The T7 RNA polymerase binding site present in the T7 primer was used for *in-vitro* transcription. The T7 polymerase initiates RNA synthesis at the “gggaga” sequence motif of the promoter region and thus the 5′ end of the aptamer sequence contains only 19 nucleotides of the conserved primer binding site (Figure 1A). The SP6 primer contributes 45 nucleotides to the 3′ end of the aptamer sequence (Figure 1A). The predicted secondary structure of the four selected RNA aptamers, Apt16, 25, 68 and 79, indicates the presence of complex structural elements such as stem-loop regions (Figure 1B–E).

**Figure 2:**
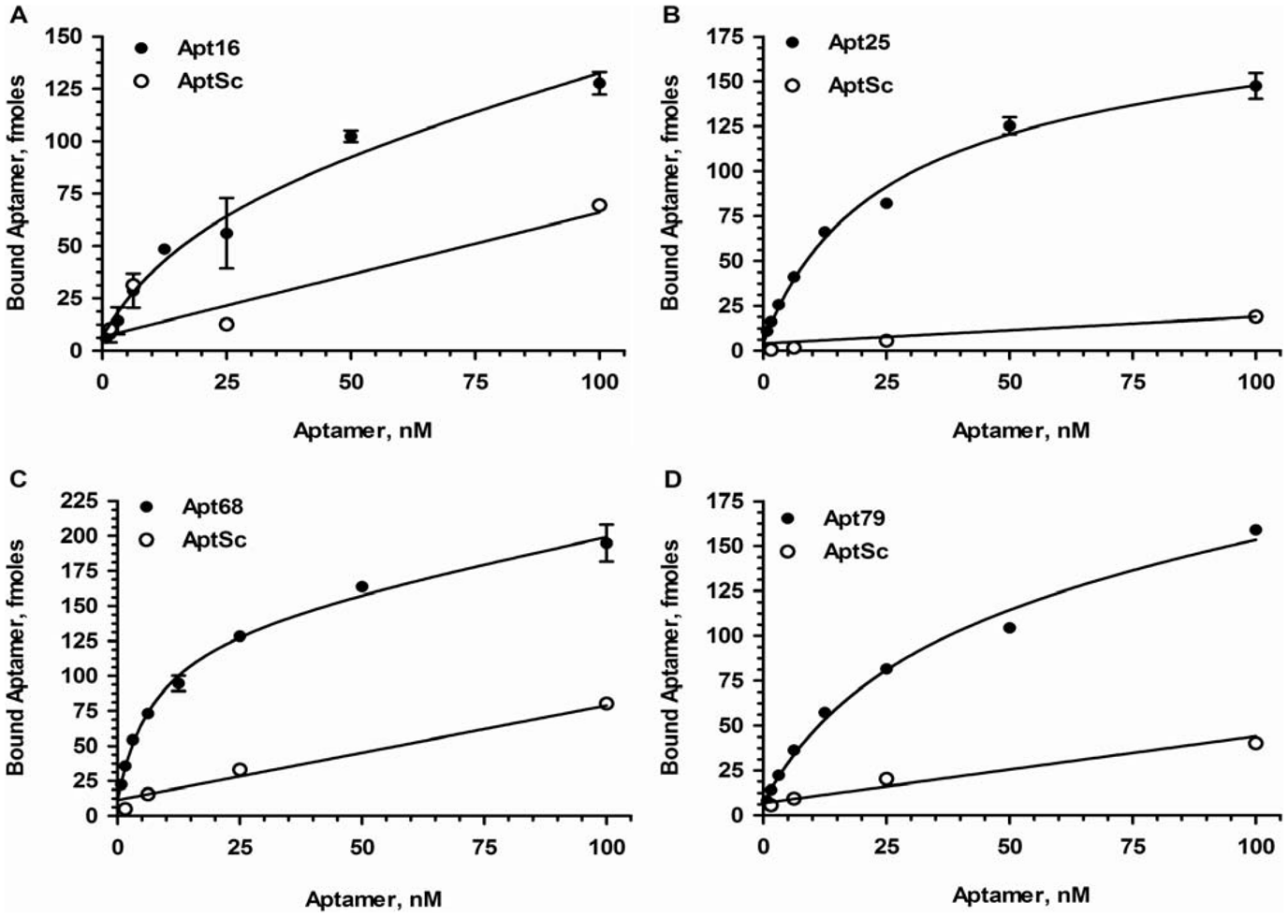
Binding analysis of aptamers with *T. cruzi* trypomastigotes. A representative clone (Apt16, 25, 68 and 75) from each aptamer family, obtained at round 12 of SELEX was used in radiolabeled binding studies. ^32^P-GTP labeled monoclonal aptamers at varying concentration beginning at 100 nM were incubated with a fixed number of trypomastigotes (2×10^6^ parasites) in a 200 µl reaction. After the parasites were washed with SELEX buffer, the amount of aptamers bound to the parasites were determined as described in Materials and Methods. Specific activity of the labeled aptamer was used to calculate the fmole amount of aptamer bound to the parasites. Each data point represents triplicate values and the error bars represent the standard deviation. Apt16 (A), Apt25 (B), Apt68 (C) and Apt79 (D) all showed dose depended saturable binding to the parasites while the respective scrambled controls showed linear non-specify binding. The saturating curve obtained was analyzed using GraphPad Prism 5, under the non-linear fit model (global fitting of total and nonspecific), with specific (aptamer) and nonspecific (scrambled aptamers, Apt Sc) binding, and Bmax and Kd were calculated (Table 1).

One representative sequence from each family described above was used in binding assays with radiolabeled aptamers to determine their binding characteristics to live trypomastigotes. For binding experiments, mononucleotide scrambled aptamers were used as non-specific binding controls. The nucleotide composition of the test and control aptamers are the same, however, due to the scrambled primary sequence of the control aptamers, they will not fold into the exact same tertiary structure as the test aptamers and therefore will not bind to the target molecule. Thus mononucleotide shuffled sequences are the best controls to demonstrate the binding specificities of aptamers [17]. Two fold serial dilutions of test and control aptamers, beginning at the highest concentration of 100 nM, were incubated with 2×10^6^ trypomastigotes in duplicate. After incubation the cells were washed 3 times with SELEX buffer. The amount of radiolabeled aptamers (in fmoles) associated with each pellet fraction was estimated based on the specific activity of the labeled RNA. The four selected aptamers, Apt16, 25, 68 and 79, showed saturable binding to live trypomastigotes while the control aptamers showed low or negligible binding (Figure 2). It is interesting to note that the Kd values for these aptamers were in the low nM range (Table 1). None of the scrambled aptamers bound significantly to the parasites indicating that the primary sequence and hence, the tertiary structures of the aptamers were crucial for their binding activity. To obtain aptamers that would bind to targets present at very low concentrations, the affinities of the aptamers have to be in low to sub-nanomolar range and thus the SELEX conditions to increase stringency utilized high volume wash steps (Table S1) [18]. The use of chaotropic agents or high salt in the wash buffer to increase stringency of aptamer-target interaction was not attempted as it would affect the viability of the parasites. Additionally, to prevent internalization of surface bound aptamers, the SELEX and binding experiments were performed on ice.

**Table 1:** Binding characteristics of the selected aptamers.

Aptamer	Kd (nM)	Bmax (fmoles)	Estimated number of Binding sites/parasite
Apt68	7.62±1.63	129.3±7.28	20138
Apt25	22.12±3.9	157.4±11.05	24515
Apt16	22.96±1.6	81.77±23.01	12615
Apt79	29.92±5.62	142.10±11.83	22116

Fixed concentrations of trypomastigotes were incubated with serial dilutions of radiolabeled aptamers, Apt16, 25, 68 and 75 (Figure 2). The saturation curve obtained was analyzed using GraphPad Prism5 software under the non-linear fit model (global fitting of total and nonspecific), with specific (aptamer) and nonspecific (scrambled aptamer) binding, and Bmax and Kd were calculated and shown in this table. The estimated number of binding sites per parasite was calculated as explained in the Methods section.

Other SELEX strategies have been used to generate aptamers against trypanosomatid parasites. For example, SELEX for aptamers that bind cell adhesion molecules expressed on the surface of *T. cruzi* trypomastigotes was performed with the aim to develop inhibitors of host-parasite interaction [19]. Aptamers that were bound to the parasite were eluted using heparin sulfate and other adhesion molecule agonists. Although this approach yielded aptamers that bound to trypomastigotes, their binding affinities to *T. cruzi* trypomastigotes were considerably higher (40–400 nM) than those obtained in our study and thus would have limited utility as ligands for parasite concentration from dilute suspensions [19]. A different approach, using recombinantly expressed variant surface glycoprotein as a target for SELEX, resulted in aptamers that bound live *T. brucei* trypomastigotes [20], [21]. A similar strategy could be applied to *T. cruzi* and yield high affinity aptamers, however, our approach was to screen for aptamers using live trypomastigote cells rather than utilize a single protein as a target. In addition our goal was to generate multiple aptamers that could potentially bind to different targets on whole cells thus providing some redundancy in assays where the aptamers would be used as ligands for parasite concentration.

### Apt68 Interacts with High Specificity to *T. cruzi* Trypomastigotes

Of the aptamers tested in the initial binding assays, Apt68 demonstrated the highest affinity (Kd 7.68±1.63 nM). Based on the Bmax and the number of parasites used in the assay, the number of binding sites was estimated to be ≥20,138/cell (Figure 2, Table 1). As Apt68 had the best binding characteristics, based on the large number of binding sites per parasite and nanomolar range high affinity, it was chosen for specificity studies. The binding of Apt68 was specific as the mononucleotide scrambled aptamer did not bind to trypomastigote cells (Figure 2C). Since both the scrambled aptamer and Apt68 encode the constant flanking regions contributed by the T7 (1–19 nucleotides) and SP6 (74–118 nucleotides) primers, the sequence responsible for parasite binding is more likely to be present in the random region. In order to investigate the possible contribution of the conserved flanking region to parasite binding we generated a truncated version of Apt68. Since the T7 region comprising 1–19 nucleotides can not be deleted as they are essential for in-vitro transcription, the deletion was made in the SP6 primer. SP6 primer with 27 base deletions at the 5′ end was synthesized and used for PCR amplification of the Apt68 clone. The purified PCR product was used for *in-vitro* transcription yielding an aptamer Apt68-del composed of 91 nucleotides compared to the full length Apt68 with 118 nucleotides (Figure S2A). Secondary structure prediction indicated that the primer deletion resulted in the loss of two stem-loop structures at the 3′ end of the aptamer (Figure S2, B, C and D, shaded regions). However, this deletion did not seem to change the predicted secondary structure of the remaining aptamer sequence significantly. Binding studies showed that Apt68-del bound to trypomastigotes with a lower Bmax (87±2.67 fmoles) compared to full length Apt68 (151.41±4.87 fmoles). As the Apt68-del bound with high affinity (10.08±1.68 nM) to the trypomastigotes, this suggests that the SP6 region of the aptamer is needed but not essential for optimal binding (Figure S2E).

**Figure 3:**
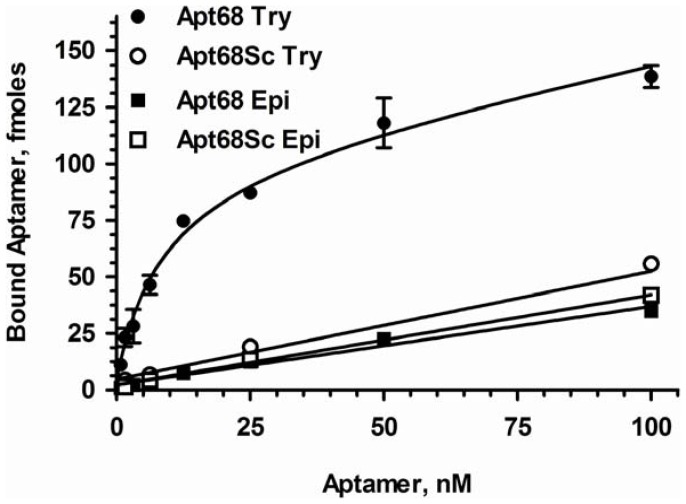
Apt68 binds to *T. cruzi* mammalian trypomastigote stage and not to the insect epimastigote stage. ^32^P-GTP labeled Apt68 was used in binding assays as in Figure 2. Apt68 showed dose depended saturable binding only to the mammalian stage trypomastigotes and not to the insect stage epimastigotes. The control, Apt68 Sc, did not bind to either of the parasite life cycle stages. Each data point represents triplicate values and the error bars represent the standard deviation. As Apt68 did not show any significant binding to epimastigotes, above that of the scrambled aptamer, Bmax and Kd could not be calculated.

**Figure 4:**
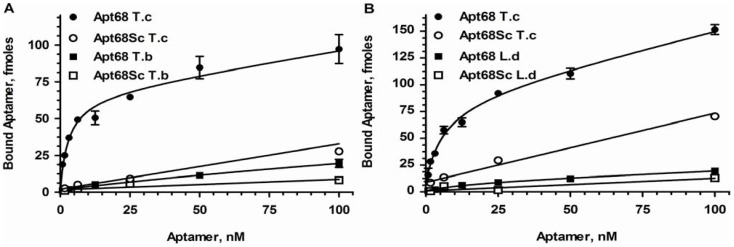
Binding of Apt68 is specific to *T. cruzi* and not to other related parasites, *T. brucei* and *L. donovani.* ^32^P-GTP labeled Apt68 was used in binding assays as in Figure 2. Apt68 is highly specific for the mammalian stage of the parasite and showed dose depended saturable binding compared to *T. brucei* blood stream form (A). Apt68 also did not interact significantly with *L. donovani* promastigotes (B). Each data point represents triplicate values and the error bars represent the standard deviation. As Apt68 did not show significant binding to the *L. donovani* and *T. brucei* parasites, above that of the scrambled aptamer, Bmax and Kd could not be calculated.

To further demonstrate binding specificity of Apt68 to trypomastigotes, binding assays were performed with the insect stage epimastigotes of the parasite. As *T. cruzi* is dimorphic, showing two distinct life cycle stages, with trypomastigotes present in infected mammalian hosts and epimastigotes in insect vectors, lack of binding to epimastigotes would indicate a high degree of specificity [22]. Results indeed showed that there was negligible binding of Apt68 and its scrambled control to epimastigotes, indicating that the target of Apt68 is expressed only by the mammalian stage trypomastigotes (Figure 3). The Kd for interaction with epimastigotes could not be calculated due to the lack of significant binding over the background scrambled aptamer. To further evaluate the binding specificity of the aptamer, binding assays were performed with two other trypanosomatid parasites, *Leishmania donovani* and *Trypanosoma brucei*. Apt68 did not bind to *L. donovani* promastigotes or the *T. brucei* blood stream forms and thus showed specificity only to *T. cruzi* trypomastigotes (Figure 4A, B). A major challenge for serological testing of Chagas disease is the high cross reactivity seen in patient sera with Leishmaniasis, African Trypanosomiasis and other parasitic diseases [23], [24]. This phenomenon has led to the high rates of false positivity seen in Chagas diagnostic assays [23], [24]. Thus, the specificity exhibited by Apt68 in recognizing targets only on *T. cruzi* trypomastigotes may be important for diagnostic applications.

### Apt68 Coated Magnetic Beads Bind to Live *T. cruzi* Trypomastigotes

**Figure 5:**
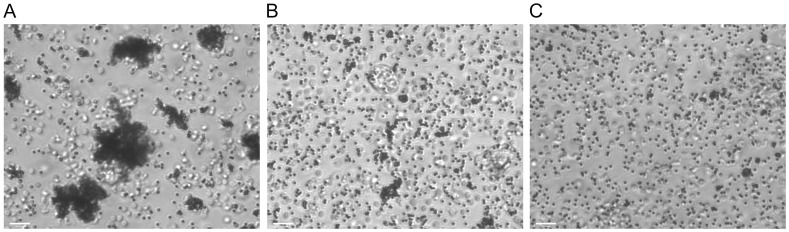
Interaction of biotinylated aptamer coated streptavidin paramagnetic beads with *T. cruzi* trypomastigotes. Streptavidin beads coated with 100 nM of biotinylated Apt68 or the scrambled control aptamer, Apt68 Sc, or plain beads were incubated with trypomastigotes in SELEX buffer. Aggregates of beads bound to parasites were observed only in the presence of Apt68 coated beads and trypomastigotes (A). Scrambled aptamer (Apt68 Sc) coated beads (B) or beads alone (C) did not cause aggregation when incubated with trypomastigotes.

The results above indicated that free Apt68 could bind to *T. cruzi* trypomastigotes. In order to determine if this binding activity would be retained when the aptamer was immobilized on a solid phase, streptavidin paramagnetic beads coated with biotinylated Apt68 was incubated with live parasites. The assay was designed to microscopically visualize aggregates of parasites and magnetic beads that would form only in the presence of Apt68. Live parasites and Apt68-coated beads were mixed in the presence of 80% human plasma and incubated at room temperature for 30 minutes in a 96 well flat bottom plate. As the assay was performed with live flagellated trypomastigote forms of the parasite, large “motile” aggregates could be detected as early as five minutes after mixing the aptamer coated beads with the parasites (Figure 5A, Supplement Movie S1). These motile aggregates of parasites and beads were found to be stable even after being incubated at 37°C for 36 hours (data not shown). No aggregation was observed in the wells where biotinylated scrambled aptamer was used (Figure 5B) or when trypomastigotes were mixed with beads alone (Figure 5C). Aggregates were also not formed when the Apt68 coated beads were incubated with live *T. cruzi* epimastigotes (Movie S2). Additionally, no aggregates were observed when the aptamer coated beads were incubated with red blood cells (RBCs) or alone in the absence of parasites. In order to verify that the incorporation of biotin does not disrupt Apt68 structure or binding to its target on the cell surface of trypomastigotes, radiolabeled Apt68 with and without the incorporated biotin-11-ATP was used in binding studies as described above. The presence of a 11 carbon linker between the nucleotide and biotin allows for enhanced flexibility and thus minimizes stearic hindrance or significant disruption of the aptamer structure. Due to the presence of 28 adenine residues in the sequence of Apt68, and based on the molar ratio of 1∶10, Biotin-11-ATP: ATP, the aptamer is modified by 2 to 3 biotin per molecule of the aptamer. Thus, we expect that the low number of modified nucleotides incorporated during *in-vitro* transcription will not drastically affect the binding properties of the biotinylated Apt68. Results showed that the biotinylated aptamer bound with high affinity (32.68±11.89 nM) to trypomastigotes (Figure S3). However, compared to the non-biotinylated Apt68, there was a decrease in affinity. Nevertheless, as each individual bead is coated by multiple aptamer molecules, resulting in a high avidity interaction between the beads and parasites, this decrease in aptamer affinity was acceptable. This was evident by the presence of large clusters of beads bound to parasites in aggregation assays (Figure 5A, Supplement Movie S1). Additionally, the incorporation of biotin during *in-vitro* transcription allowed for large scale synthesis of biotinylated Apt68. Other methods of RNA biotinylation have been described in literature, however, biotin incorporation during *in-vitro* transcription of Apt68 provided significantly higher yield (20–40 times higher) compared to an end labeling method (data not shown) [25], [26], [27].

To demonstrate that the aptamer bound to a cell surface ligand, trypomastigotes treated with trypsin were incubated with Apt68 coated beads. No aggregates were observed in trypsin treated trypomastigotes (Movie S3) while untreated trypomastigotes formed motile aggregates as before (Movie S1). These results indicate that Apt68 interaction with the parasite is highly specific. Apt68 bound to parasites even in the presence of 80% plasma. This indicated that Apt68 could be useful in assays where parasites could be captured from *T. cruzi* contaminated blood in the presence of all potential interfering agents. Additionally, as the aggregates were stable for >36 hours in 80% plasma, this suggested that modified fluorinated deoxynucleotides 2′ F-dUTP and 2′ F-dCTP, utilized during the *in-vitro* transcription helped in generating nuclease resistant aptamers. The absence of aggregates in parasites treated with trypsin confirmed that Apt68 bound to a surface expressed target that includes a trypsin sensitive peptide component. Trypsin treatment did not change the viability of the parasites as >95% were still highly motile. Taken together, these results show that Apt68 coated magnetic beads bind to cell surface of live trypomastigotes and this interaction was highly specific to a trypomastigote expressed target.

Since the SELEX was performed with trypomastigote forms of a laboratory strain of *T. cruzi* (Tulahuen), it was essential to demonstrate that Apt68 would also bind to trypomastigotes from other parasite strains. To this end, epimastigotes obtained via hemoculture from 5 seropositive blood donors, comprising the two major *T. cruzi* genotypes, I and II, and the laboratory Y2 strain of the parasite, were grown to stationary phase in order to induce differentiation of the epimastigotes into infective metacyclic trypomastigotes [28], [29]. These stationary phase cultures were used to infect a monolayer of murine 3T3 cells. All the parasite isolates tested, were able to infect 3T3 cells and differentiate into amastigotes. After 2–3 weeks of culture, mixed populations of 3T3 cell derived trypomastigotes and epimastigote-like forms were seen in culture supernatants as reported before [30]. However, as repeated passages continued to give mixed cultures of trypomastigotes and epimastigote-like morphologies, and due to the inability to purify trypomastigotes, quantitative binding assays with radiolabeled Apt68 could not be performed and only qualitative microscopic evaluation of aggregate formation was attempted. Nevertheless, as in the laboratory Tulahuen strain of the parasite, motile aggregates of trypomastigotes and beads were observed in all the parasite clones tested (movie shown only for isolate 0704 (Type I), Movie S4). No aggregation of beads with the epimastigote-like forms was seen. The distinction between trypomastigotes and epimastigote-like forms in the mixed cultures was made based on the characteristic motility and morphologies of the two forms (Movie S4). Although aggregation assays with tissue culture derived trypomastigotes from blood donor isolates were successful, these trypomastigotes may be functionally distinct from blood stage trypomastigotes seen in patients. Nevertheless, these experiments do indicate that the target of Apt68 was structurally conserved among various strains and genotypes of *T. cruzi* and that Apt68 could have potential applications in clinical settings.

### Aptamer Based *T. cruzi* Trypomastigote Concentration from Spiked Samples

**Figure 6:**
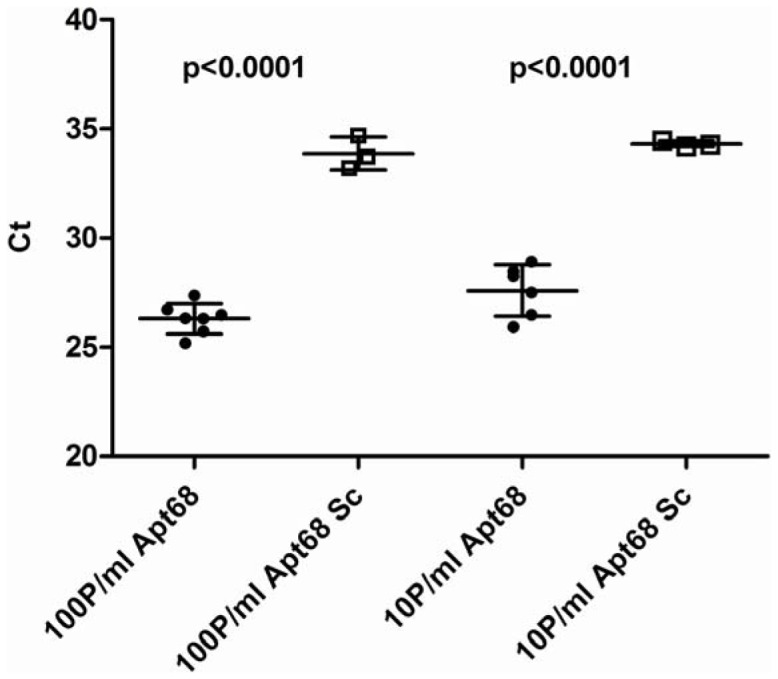
Pull down assay with *T. cruzi* trypomastigotes from spiked plasma samples. Streptavidin beads, loaded with Apt68 or scrambled aptamer (Apt68 Sc) prepared in SELEX buffer were incubated with a suspension of *T. cruzi* trypomastigotes, prepared in human plasma, at a concentration of 100 parasites/ml or 10 parasites/ml. The parasite-bead aggregates were then concentrated using a magnet and washed with SELEX buffer. DNA was extracted using the Direct Lysis Buffer (Sigma, Mo.) and aliquots used for real time PCR. The data plotted is a representative of 3 independent experiments. Mean Ct values are plotted on the y-axis with error bars representing the standard deviation of 7 replicates. There was a significant difference (p<0.001) between the amount of parasite DNA amplified from the samples, when Apt68 coated beads were used, compared to the scrambled aptamer coated beads, low Ct values indicating higher DNA concentration. This trend is observed at both concentrations of the spiked parasites, 100 parasites/ml and 10 parasites/ml. Negative controls, where nuclease free water was used for amplification, showed Ct >40.

Given that Apt68 showed trypomastigote binding activity, even when immobilized on a solid phase, and that Apt68 coated paramagnetic beads could form aggregates with all the strains tested thus far, the next goal of this study was to determine if these beads could be used to pull down and concentrate parasites from samples, which had low levels of parasites, and facilitate their detection by PCR. To assess the feasibility of this approach, Apt68 coated paramagnetic beads were first incubated with trypomastigote spiked plasma samples. The parasites were spiked at a concentration of 100 and 10 trypomastigotes/ml (parasites/ml, p/ml) in a fixed volume of 200 µl plasma. After incubation on ice for 30 minutes, the parasite-bead aggregates were concentrated using a magnet. The supernatant was discarded and the pellet was washed in SELEX buffer. The aggregates were concentrated again and the pellet was resuspended in DNA direct lysis buffer. Aliquots of the lysed samples were used in real time PCR assays to detect *T. cruzi* DNA. The assay was independently repeated three times and data from a representative assay with 7 replicates of 100 or 10 parasites/ml was analyzed to obtain the average Ct values (Figure 6). The samples where Apt68 was used for parasite pull down showed a significantly lower Ct (p<0.001), Ct = 26.33±0.7 for 100 p/ml and 28.00±1.2 for 10 p/ml, compared to scrambled controls, Ct = 33.88±0.75 for 100 p/ml and 34.20±0.13 for 10 p/ml, indicating the presence of higher amounts of parasite genomic DNA. The fact that samples pulled down using scrambled aptamer coated beads showed amplification, indicated that some parasites may bind non-specifically to the scrambled aptamers, as also observed in the radioactive binding assays, or that this non-specific binding may be due to incomplete washing of the beads after parasite capture. In any event, Apt68 coated beads were able to concentrate parasites from human plasma at low cell densities.

**Figure 7:**
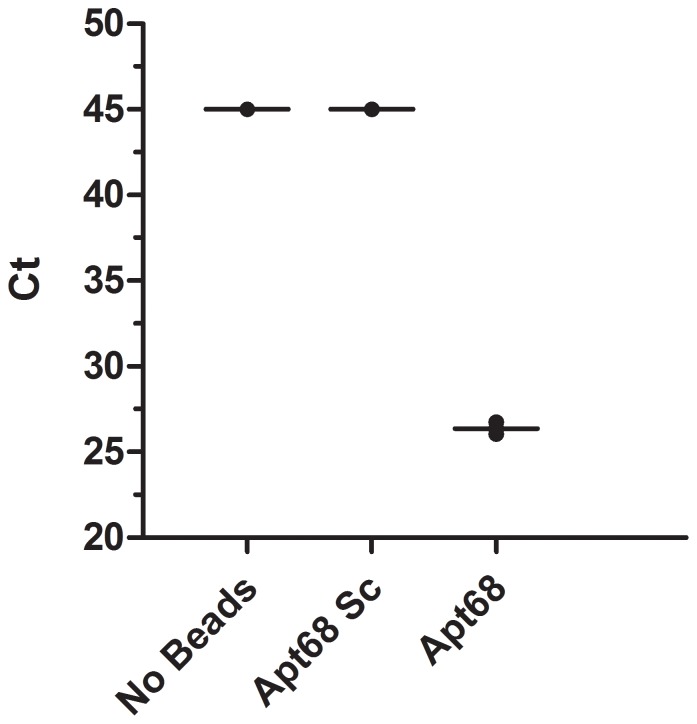
Pull down assay with *T. cruzi* trypomastigote spiked human whole blood samples. Human whole blood samples (15 ml) spiked with trypomastigotes at a concentration of 0.33 parasites/ml (5 parasite in 15 ml) was subjected to the pull down using Apt68 coated paramagnetic beads as described in the Methods section. The aptamer coated beads were incubated, for 1 hour at room temperature, with the parasite spiked whole blood samples. The parasite-bead aggregates were recovered using a magnetic stand suitable for 15 ml falcon tubes. After 3 washes, with 15 ml SELEX buffer per wash, the bound beads were recovered from the magnet, genomic DNA isolated and amplified in the real time PCR assay. The whole blood pull down assay, using blood from 3 different donors, was repeated in 5 independent experiments and data from a representative experiment is shown. Mean Ct values are plotted on the y-axis with error bars representing the standard deviation of 2 replicate PCR tests. Results showed that when Apt68 was used in the pull down assay, the Ct value was significantly lower than that obtained for samples treated with either the scrambled aptamer (Apt68 Sc) or in the absence of any beads. Based on the negative controls of the real time PCR assay, where only nuclease free water was used as an amplification template, a sample with Ct values higher than 40 cycles was considered as a negative.

Next, in order to determine if *T. cruzi* parasites present at low parasitemia (<1 parasite/ml) in blood could also be concentrated using Apt68 coated paramagnetic beads, human whole blood was spiked with trypomastigotes at a concentration of 0.33 parasites/ml (5 parasite in 15 ml) and subjected to the pull down and PCR as described above. This assay was independently repeated 5 times using blood from 3 different donors and data form a representative experiment is shown in Figure 7. Results showed that when Apt68 was used in the pull down assay, the Ct value of the sample was significantly lower than that obtained when either the scrambled aptamer was used or in the absence of any beads (Ct = 25±0.2 vs. Ct = 45 and Ct = 45, respectively) (Figure 7). In all the pull down experiments, genomic DNA samples from scrambled aptamer coated beads did not show significant Ct value (Ct>45). Based on additional negative controls of the real time PCR assay, where only nuclease free water was used as an amplification template, a Ct higher than 40 cycles was considered as a negative sample. To confirm that the parasitemia in the spiked whole blood samples were indeed quite low, <1 parasite/ml, a parallel spiked blood sample identical to the one used in the pull down experiment (5 parasites in 15 ml) was generated. From this sample twenty 200 µl aliquots were processed using the Qiagen whole blood DNA extraction kit. Real time PCR with the extracted DNA indicated that only one out of the 20 aliquots was positive for *T. cruzi* DNA. This indicated that there was at least 1 parasite present in 4 ml of the sample tested (representing 26.6% of the total 15 ml spiked blood sample); thus indicating that the parasitemia was indeed within the range of 0.33 parasites/ml. These results show that, using Apt68 coated paramagnetic beads, it was possible to concentrate parasites spiked in human whole blood at a concentration of 0.33 parasites/ml.

The concentration of *T. cruzi* trypomastigotes from whole blood using Apt68 coated magnetic beads prior to Qiagen Blood DNA extraction described above offers advantages over a direct DNA extraction. For example, the bead capture process allows for the removal of well known PCR inhibitors, such as host genomic DNA and heme from lyzed RBCs, which are co-purified along with parasite DNA using Qiagen or similar commercial kits [31], [32]. Further, the aptamer-based pathogen concentration method may help facilitate the detection of parasites in clinical specimens with low parasitemia such as in specimens collected either, during the early phase of infection (the window period of PCR detection), or during the chronic phase when there is intermittent parasitemia in blood [5], [33]. Current assays that detect *T. cruzi* genomic DNA require a sample from 2–20 ml of blood to be lyzed and analyzed by PCR [5], [32]. Since Apt68 retains its binding activity when immobilized to a solid phase, it is conceivable that filtration devices incorporating this aptamer as a parasite specific ligand could allow the testing of a whole unit of blood from a donor. This parasite reduction approach would provide an additional layer of safety, when used concurrently with approved serological tests. Thus, aptamer based *T. cruzi* capture devices may assist in the non-destructive analysis of blood donations, by simultaneously capturing, as well as, facilitating pathogen detection from large volumes of blood. In this scenario, sero-negative and PCR negative units would be used for transfusion. Recent work on the capture and detection of cancer cells from blood, using aptamer coated micro capillaries, vindicates such approaches [34].

In summary, our results provide a proof-of-concept for the use of aptamers to concentrate parasites from blood samples and their potential to facilitate pathogen detection by PCR based assays. The validation of Apt68 as a ligand for *T. cruzi* concentration and reduction using clinical samples will be performed in the future. Other applications such as targeted drug delivery will also be investigated.

## Materials and Methods

### Parasites

*T. cruzi* trypomastigotes, Tulahuen strain and epimastigotes obtained by hemoculture from 5 seropositive blood donors (of genotypes I and II) were kindly provided by David Leiby, American Red Cross and maintained in culture as previously described [35]. Briefly, *T. cruzi* trypomastigote parasites (Tulahuen) were allowed to infect a monolayer of murine 3T3 cells, obtained from American Type Culture Collection (ATCC; http://www.atcc.org) and cultured in 5% fetal bovine serum and IMDM. After 24 hours of incubation, the monolayer was washed with medium to remove extracellular parasites. The infected monolayer was cultured for 4–5 days till extracellular motile trypomastigotes were observed in the medium. For the Y2 strain, kindly provided by Ester Roffe Santiago, NIAID, NIH and the blood donor isolates, the epimastigotes were cultured in LIT medium till stationary phase was obtained [29]. To obtain trypomastigotes of the blood donor isolates, the stationary phase epimastigote culture containing infectious metacyclic trypomastigotes were washed and used to infect 3T3 cells. After 4–5 days of infection, the culture medium was replaced with fresh medium to remove non-infectious parasites. Thereafter the culture medium was replaced every day for one week and amastigote infected cells were cultured for an additional 2–4 weeks till extracellular trypomastigotes were observed [30]. Tissue derived trypomastigotes of blood donor isolates were passaged and maintained similar to the laboratory strain. Trypomastigotes present in culture supernatant of infected 3T3 cells was collected by centrifugation, counted using a hemocytometer and used for binding and aggregation assays. For specificity studies *Leishmania donovani* promastigotes and *T. brucei* blood stream forms were cultured as described before [36], [37], [38]. The cells were pelleted, washed with PBS and counted using a hemocytometer.

### Oligonucleotide Synthesis

Oligonucleotide library was prepared commercially from Midland Scientific, Texas. The DNA library used in this study had the following sequence 5′ ccggaattcatttaggtgacactatagaagtgtcgtgcattctta (N)_80_ accgttaccattatctccctatagtgagtcgtattaggatccgag 3′ in which N has an equal probability of being each of the four nucleotides. The library was amplified using the forward SP6 primer 5′ ccggaattcatttaggtgacactatagaagtgtcgtgcattctta 3′ containing an *Eco*RI restriction site (underlined) and the T7 reverse primer 5′ ctcggatcctaatacgactcactatagggagataatggtaacggt 3′ containing a T7 polymerase binding site and a *Bam*HI restriction site (underlined). The library was amplified using PCR with F- Prime Taq DNA polymerase using manufacturer suggested protocol. The conditions for the PCR were standardized to include a final concentration of 1 µM T7 forward and SP6 reverse primer, with 1 mM MgCl_2_. The amplification was carried out at an annealing temperature of 65°C for 30 seconds, followed by 72°C for 30 seconds. The numbers of cycles were limited to 20 to prevent spurious amplicons from arising. The template used for initial amplification was 20 nM in a final volume of 5 ml (∼1 × 10^14^ unique sequences). The PCR product was ethanol precipitated and purified over a G-50 sephadex micro column. The final concentration of the library was adjusted to 1 µg/µl. For the initial RNA aptamer pool the transcription was carried out using the Durascribe 2′ F-dUTP and 2′ F-dCTP (fluorinated deoxynucleotides), *in-vitro* T7 polymerase transcription kit from Epicenter Biotechnologies using manufacturer protocol, with 1 µg of the library as template. Based on the yield, the diversity was estimated to be >10^12^ unique sequences. The library was cloned and 100 individual clones sequenced to identify sequences. Only unique sequences were found in the sample. For aptamer deletion experiments shown in Figure S2, the Apt68 plasmid clone was amplified by using the SP6-del forward primer 5′ aagtgtcgtgcattctta 3′ and the T7 reverse primer described above. The deletion in the SP6-del primer resulted in the RNA aptamer length being reduced by 27 nucleotides compared to full length.

### Whole Cell SELEX

Selection for trypomastigote specific aptamers was performed using tissue culture derived trypomastigotes from the laboratory adapted Tulahuen strain of *T. cruzi*. The aptamer pool was refolded in incomplete IMDM media (SELEX buffer), without any fetal bovine serum, by heating at 65°C and then allowing the solution to equilibrate to room temperature over a 30 minute incubation. Parasites were incubated with the refolded aptamers and then washed to remove unbound and weakly bound RNA molecules. The incubations were performed on ice to prevent internalization of surface bound aptamers. Bound RNA was extracted from the cell pellet using the Ambion total RNA purification kit. The extracted RNA was reverse transcribed into DNA and amplified as described above. The amplified DNA was then used to generate serum stable RNA library using the Epicenter Durascribe T7 *in-vitro* transcription kit as described above. Twelve rounds of iterative selection were performed. In the later rounds of selection, negative selection was performed with cellular components, including red blood cells (RBCs), collected from whole blood. The aptamer pools were incubated with 6×10^7^ RBCs for 30 minutes on ice. The suspension was centrifuged at 3000 rpm for 5 minutes at 4°C. The supernatant containing unbound aptamers was recovered and incubated with trypomastigotes. This process was repeated 4 times, before subsequent selection rounds, to remove aptamers that may have cross reactivity to cellular components of blood. Additional details of the SELEX method are provided in Table S1.

### RT-PCR, Recovery of Library and Sequence Analysis

RNA eluted from the parasites was reverse transcribed using ThermoScript, a thermo stable reverse transcriptase, using manufacturer protocol. The cDNA was then amplified using PCR conditions described above. The PCR cycles were limited to 20 to prevent over amplification. After 12 rounds of SELEX the PCR product was cloned in pCR2.1 TOPO vector and sequenced using standard ABI sequencing methods. One hundred sequences obtained were analyzed using CLC Sequence Viewer and Sequencher 2.1 (Gene Codes). Sequences with complete primer binding regions were then used for phylogenetic analysis and representative cloned of dominant families were selected for further binding analysis. RNA folding software tool in CLC Main Workbench (Ver. 6.6.2), which uses a minimum free energy (MFE) approach to predict RNA secondary structure, was used to analyze individual aptamer sequences [39].

### Parasite Binding Analysis

Trypomastigotes isolated from culture were washed and resuspended to 2×10^7^ cells/ml in the SELEX buffer, with 5% FBS and 0.1 µg/ml salmon sperm DNA. Aptamers selected for analysis were labeled using 10 µCi ^32^P-GTP in an *in-vitro* transcription reaction. For the simultaneous labeling of aptamer sequence with ^32^P-GTP and Biotin, Biotin-11-ATP was also added to the *in-vitro* transcription reaction at a molar ration of 1∶10 ATP. Free un-incorporated labeled nucleotides were removed using the sephadex G25 spin columns (Amersham GE). The specific activity of the labeled aptamer was determined using DE81 filter binding assay. Serial dilutions of the aptamers were prepared beginning at 100 nM. Parasite suspensions, 2×10^6^ in 100 µl aliquots, in replicates, were incubated with increasing amounts of ^32^P-GTP labeled aptamers. The binding assay was performed in the presence of 5% FBS and 0.1 µg/ml salmon sperm DNA to prevent non-specific binding. As non-specific controls for the aptamers tested in the binding assay, radiolabeled mono-nucleotide scrambled RNA molecules, with the same nucleotide composition and length as the selected aptamers, were generated by *in-vitro* transcription. Dilutions, beginning at 100 nM, of the ^32^P labeled aptamers and controls were then incubated with the parasite suspension for 30 minutes on ice in a 96 well cell culture plate. The parasites were washed three times with 200 µl of SELEX buffer and radioactivity associated with the pellet was determined by scintillation counting. The amount of aptamer bound was calculated using the specific activity of the labeled aptamers. To assess specificity of interaction between aptamer and parasites, 2×10^6^ *T. cruzi* epimastigotes, *Leishmania donovani* promastigotes or *Trypanosoma brucei* blood stream form parasites were also used under identical conditions as the trypomastigotes for the binding assay. The saturating curve obtained was analyzed using GraphPad Prism 5, under the non-linear fit model (global fitting of total and nonspecific), with specific (aptamer) and nonspecific (scrambled aptamer) binding, and Bmax and Kd were calculated.

### Parasite Aggregation Assay Using Biotinylated Aptamers and Streptavidin Paramagnetic Beads

Biotinylated aptamers were generated by incorporating Biotin-11-ATP (Perkin Elmer) during the *in-vitro* transcription reaction. Biotin-11-ATP was incorporated at a ratio of 1∶10 compared to normal ATP. Free un-incorporated Biotin-11-ATP was removed using the sephadex G25 spin columns (Amersham GE). The concentration of the purified aptamer was determined using OD 260 nm. The aptamer was diluted in incomplete IMDM medium to a final concentration of 100 nM. Refolded biotinylated aptamers, at 100 nM, concentration were incubated with 3 µl of MyOne Streptavidin Paramagnetic Beads (Invitrogen) for 30 minutes at room temperature. The beads were then incubated with 5% Fetal Bovine Serum to block non-specific binding sites. Aptamer loaded beads were then used to perform aggregation assays. Parasites resuspended in SELEX buffer or 80% human plasma, were incubated with the beads and observed under an inverted microscope. For trypsin treatment, parasites were resuspended in SELEX buffer and cell culture grade trypsin (2.5 U/ml) was added to the suspension. After 30 minute incubation, Apt68 coated beads were added and time lapse images were collected. Time lapse images stitched into movies using Volocity and exported as QuickTime animations.

### Parasite Pull Down and Sample Concentration Assays

Parasite counts, determined using a hemocytometer, were adjusted to obtain 100 and 10 trypomastigotes per ml of plasma. Streptavidin beads, loaded with aptamers or scrambled aptamers prepared in SELEX buffer, were incubated with 100 µl aliquots of the parasites for 30 minutes on ice. The tubes were then placed on a magnetic stand for 10 minutes at room temperature and the supernatant removed. The pellets, containing the beads and parasites, were lysed with 100 µl Direct Lysis buffer (Sigma Aldrich) and 1 µl aliquots were amplified by real time PCR. The primers, forward (A**S**TCGGCTGATCGTTTTCGA), reverse (AATTCCTCCAAGCAGCGGATA) and probe (TEX615/CACACACTGGACACCAAACAAC**Y**CTG/3IAbRQSp/) sequences for the real time PCR to amplify *T. cruzi* satellite DNA were modified from Piron et al. [40]. The reaction mixture contained 0.4 µM of the forward and reverse primers and 0.4 µM of the Taqman Probe, 1× Premix Ex Taq (Takara System). The samples were amplified in the thermo-cycler Bio-Rad CFX (BioRad System) with the following PCR conditions: first step (95°C for 30 seconds) and 45 cycles (95°C for 5 seconds and 64.2°C for 30 seconds). The threshold was established by negative controls where reactions set in the absence of trypomastigotes were lyzed and utilized as template. Pellets that gave a Ct value below 40 cycles were scored as positive.

The human blood used in these studies was obtained from the National Institutes of Health Clinical Center, Department of Transfusion Medicine, under an NIH IRB-approved research protocol from volunteer donors with written consent. Use of this material was approved by the FDA IRB, (Research Involving Human Subjects Committee [RIHSC] protocol #03-120B) under 45 CFR 46 101 (B) (4). Whole blood collected from donors and spiked with trypomastigotes at a final concentration of 5 parasites in 15 ml (equivalent to 0.33 parasites/ml) in a falcon tube. Streptavidin beads, loaded with refolded biotinylated Apt68, scrambled aptamer or beads alone as a negative control, were added to the parasite spiked blood and incubated at room temperature with constant mixing for 1 hour. The tubes were then placed on a magnetic stand and incubated with constant mixing. After an additional 30 minutes of incubation the blood was decanted from the tubes while still attached to the magnetic stand. The tubes were washed three times with 15 ml of IMDM media. After decanting the last wash, the tubes were removed from the magnetic stands and the beads were recovered. Total genomic DNA was purified from the bead pellet using the Qiagen Blood DNA extraction kit. Parasite DNA was amplified using 2 µl of sample per reaction, by the methods described above. A second 15 ml spiked tube with 5 parasites (0.33 parasites/ml) was prepared and used as a control. Genomic DNA from twenty 200 µl aliquots were isolated using the Qiagen Blood DNA extraction kit and 2 µl of DNA sample used for each PCR reaction. This was done to determine the total number of tubes positive for parasite DNA as a control for the number of spiked parasites.

## Supporting Information

Figure S1 **Phylogenetic analysis of sequences obtained from the** ***T. cruzi*** **trypomastigote whole cell SELEX.** Aptamer pool obtained at round 12 of the *T. cruzi* trypomastigote SELEX was cloned into a TOPO vector and one hundred individual clones isolated and sequenced. Aptamer sequences were aligned using CLC Sequence Viewer 6.4 software. Phylogenetic analysis was carried out using the CLC Sequence Viewer 6.4 software. The clustering algorithm for distance data used was Unweighted Pair Group Method using Arithmetic averages (UPGMA). Bootstrapping was performed with 1000 replicates. The families obtained were labeled as 1 thru 4. A single representative clone from each family was utilized for binding studies.
(TIF)

Figure S2 **Deletion analysis of Apt68.** Full length Apt68, Apt68-del and scrambled (Sc) sequence is depicted (A). The conserved primer sequence is shown in lower case. Predicted secondary structure of Apt68, Apt68-del and scrambled Apt68 is shown in panels B, C, and D, respectively. The secondary structure contributed by the SP6 primer regions is highlighted. ^32^P-GTP labeled Apt68 and Apt68-del shows dose dependent saturable binding to *T. cruzi* trypomastigotes (E). The ^32^P-GTP labeled scrambled Apt68 showed minimal binding to trypomastigotes. Each data point represents duplicate values and the error bars represent the standard deviation.
(TIF)

Figure S3 **Comparison of** ***T. cruzi*** **trypomastigote binding activity of Apt68 and Biotinylated Apt68.** ^32^P-GTP labeled Apt68 with and without incorporated biotin-11-ATP was used in a dose dependent binding assay with trypomastigotes. ^32^P-GTP labeled scrambled Apt68 with and without incorporated biotin-11-APT were used as corresponding controls. The scrambled aptamers did not show significant binding to *T. cruzi* trypomastigotes while both the biotinylated and non-biotinylated Apt68 bound in a dose dependent manner with high affinity. Each data point represents duplicate values and the error bars represent the standard deviation.
(TIF)

Table S1 **Parameters used for the** ***T. cruzi*** **trypomastigote whole cell SELEX.** For the first round of SELEX, trypomastigotes at 1×10^8^/ml were incubated with 80 pmoles of the random RNA pool for 1 hour on ice. The trypomastigotes were recovered by centrifugation and RNA isolated from the pellet. In subsequent rounds, conditions for binding and dissociation were gradually increased in stringency by incorporating 5% FBS and salmon sperm DNA as inhibitors of non-specific interactions. As a means of negative selection and to remove aptamers that bound to targets of host origin, from round 4 of the SELEX, the aptamer pool was pre-incubated with human whole blood (RBC, platelets, leucocytes and plasma) and fetal bovine serum. After incubation for 30 minutes, this suspension was centrifuged and the supernatant incubated with *T. cruzi* trypomastigotes. Additionally, the binding time for the aptamers to trypomastigotes was reduced, while the number of washes and the total volume of SELEX buffer used for the washes was increased. These changes in the binding and washing protocols were made so that those aptamers in the library, that had a high association rate and a low dissociation rate, were selected. Together, these changes would result in the selection of aptamers with high affinities. The SELEX was performed for a total of 12 rounds as shown in the table.
(DOC)

Movie S1 **Biotinylated Apt68 was incubated with streptavidin paramagnetic beads and parasites.** The binding of Apt68 coated beads to trypomastigotes can be seen as large motile aggregates (Movie S1), while there are no aggregates seen in the presence of epimastigotes (Movie S2). The formation of motile aggregates occurred within the first 5 minutes of the incubation period, with larger aggregates seen by 30 minutes of incubation. All the parasites were captured by the beads and very large aggregates were seen after >24 hours of incubation (data not shown). In the movie, the beads are visible as black spheres while parasites appear as translucent motile forms.
(MOV)

Movie S2 **See Movie S1.**
(MOV)

Movie S3 **Trypomastigotes were incubated in the presence of trypsin for 30 minutes at room temperature.** Apt68 coated paramagnetic beads were added to the suspension and observed under the microscope. No aggregates could be seen even after several hours of incubation. The trypsin treated trypomastigotes were motile during this incubation period indicating that this treatment did not kill them. The untreated control parasites formed motile aggregates in the presence of Apt68 coated beads as seen before (Movie S1). As no aggregates were visible in the trypsin treated parasites, this indicated that the target of Apt68 is expressed on the cell surface of trypomastigotes. The beads are visible as black spheres while parasites appear as translucent motile forms.
(MOV)

Movie S4 **Parasites from the clinical isolate 0704 formed aggregates in the presence of Apt68 coated beads.** A mixed culture of trypomastigotes and epimastigote-like forms can be seen, where the characteristic motilities of the two populations, is easily distinguishable (more active tumbling motion of the trypomastigotes vs. the less motile epimastigote-like forms). The motile parasite-bead aggregates are visible only with the trypomastigotes and not with the epimastigote-like forms. The beads are visible as black spheres while parasites appear as translucent motile forms.
(MOV)
