# Supplementary figures and images for: Development of an Aptamer-Based Concentration Method for the Detection of Trypanosoma cruzi in Blood
Source: PLoS One. 2012 Aug 22;7(8):e43533. doi: 10.1371/journal.pone.0043533 (PMC3425475; doi:10.1371/journal.pone.0043533)

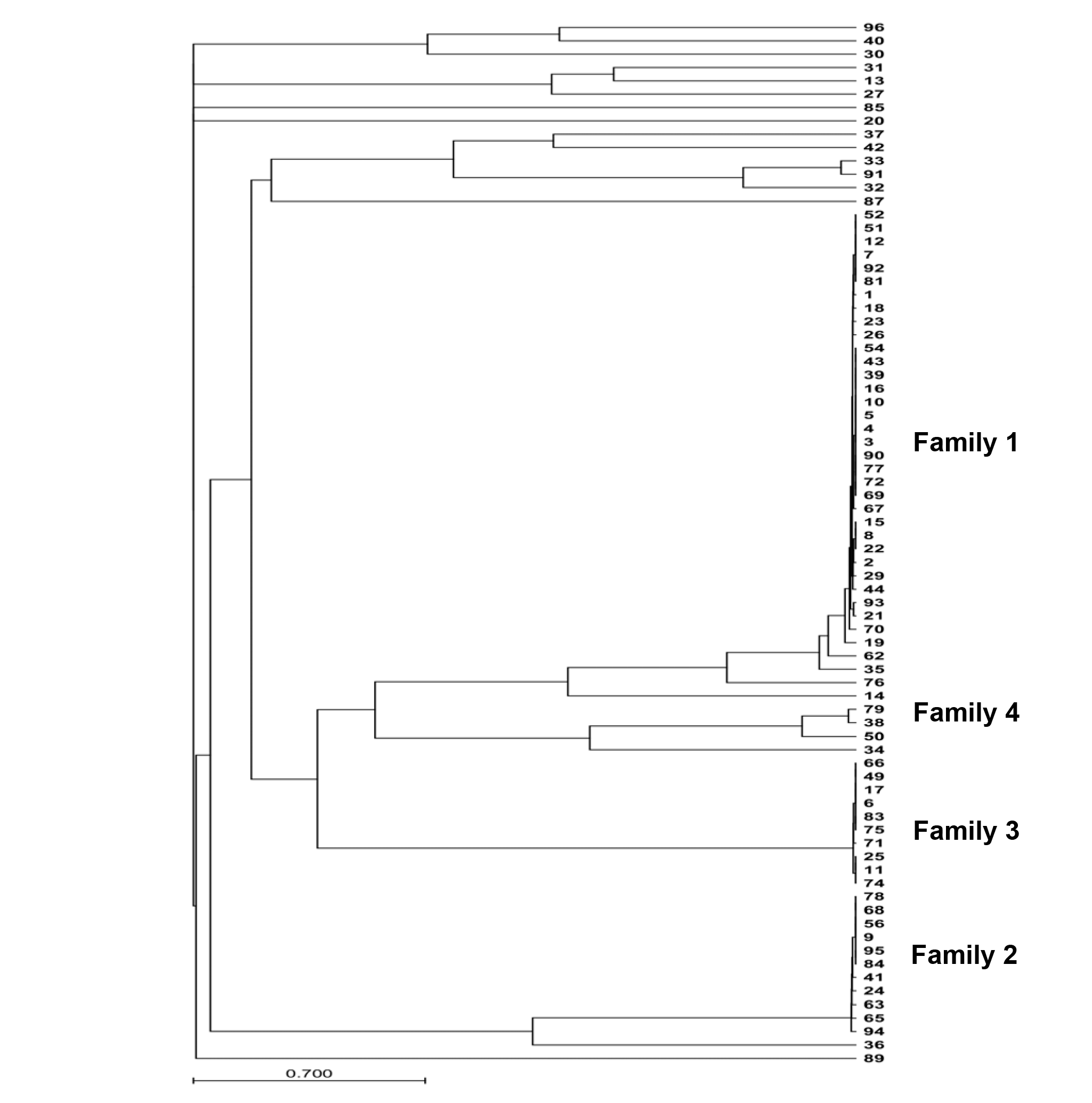

Supplement: Figure S1 — Phylogenetic analysis of sequences obtained from the T. cruzi trypomastigote whole cell SELEX. Aptamer pool obtained at round 12 of the T. cruzi trypomastigote SELEX was cloned into a TOPO vector and one hundred individual clones isolated and sequenced. Aptamer sequences were aligned using CLC Sequence Viewer 6.4 software. Phylogenetic analysis was carried out using the CLC Sequence Viewer 6.4 software. The clustering algorithm for distance data used was Unweighted Pair Group Method using Arithmetic averages (UPGMA). Bootstrapping was performed with 1000 replicates. The families obtained were labeled as 1 thru 4. A single representative clone from each family was utilized for binding studies. (TIF) [file pone.0043533.s001.tif]

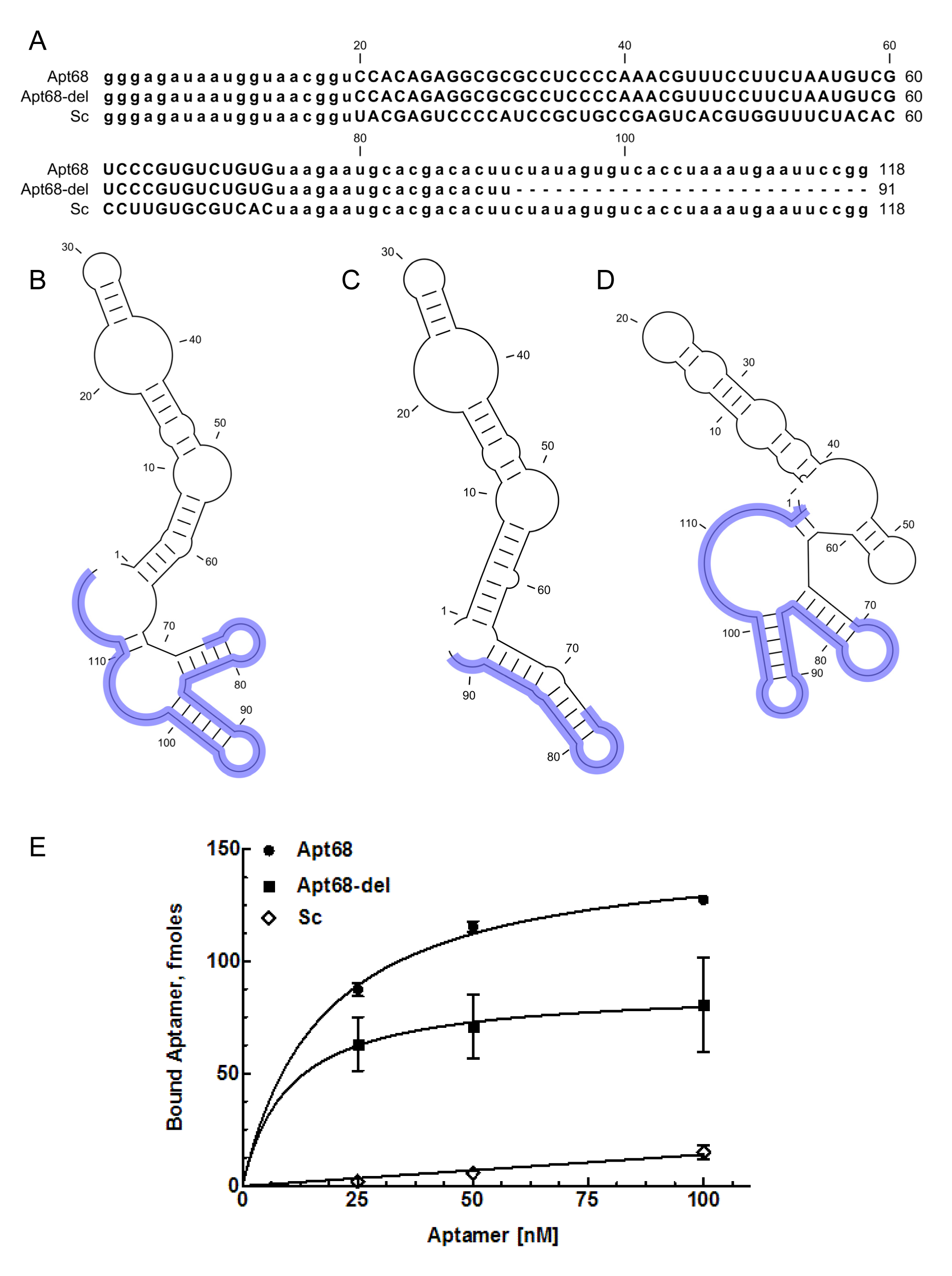

Supplement: Figure S2 — Deletion analysis of Apt68. Full length Apt68, Apt68-del and scrambled (Sc) sequence is depicted (A). The conserved primer sequence is shown in lower case. Predicted secondary structure of Apt68, Apt68-del and scrambled Apt68 is shown in panels B, C, and D, respectively. The secondary structure contributed by the SP6 primer regions is highlighted. 32P-GTP labeled Apt68 and Apt68-del shows dose dependent saturable binding to T. cruzi trypomastigotes (E). The 32P-GTP labeled scrambled Apt68 showed minimal binding to trypomastigotes. Each data point represents duplicate values and the error bars represent the standard deviation. (TIF) [file pone.0043533.s002.tif]

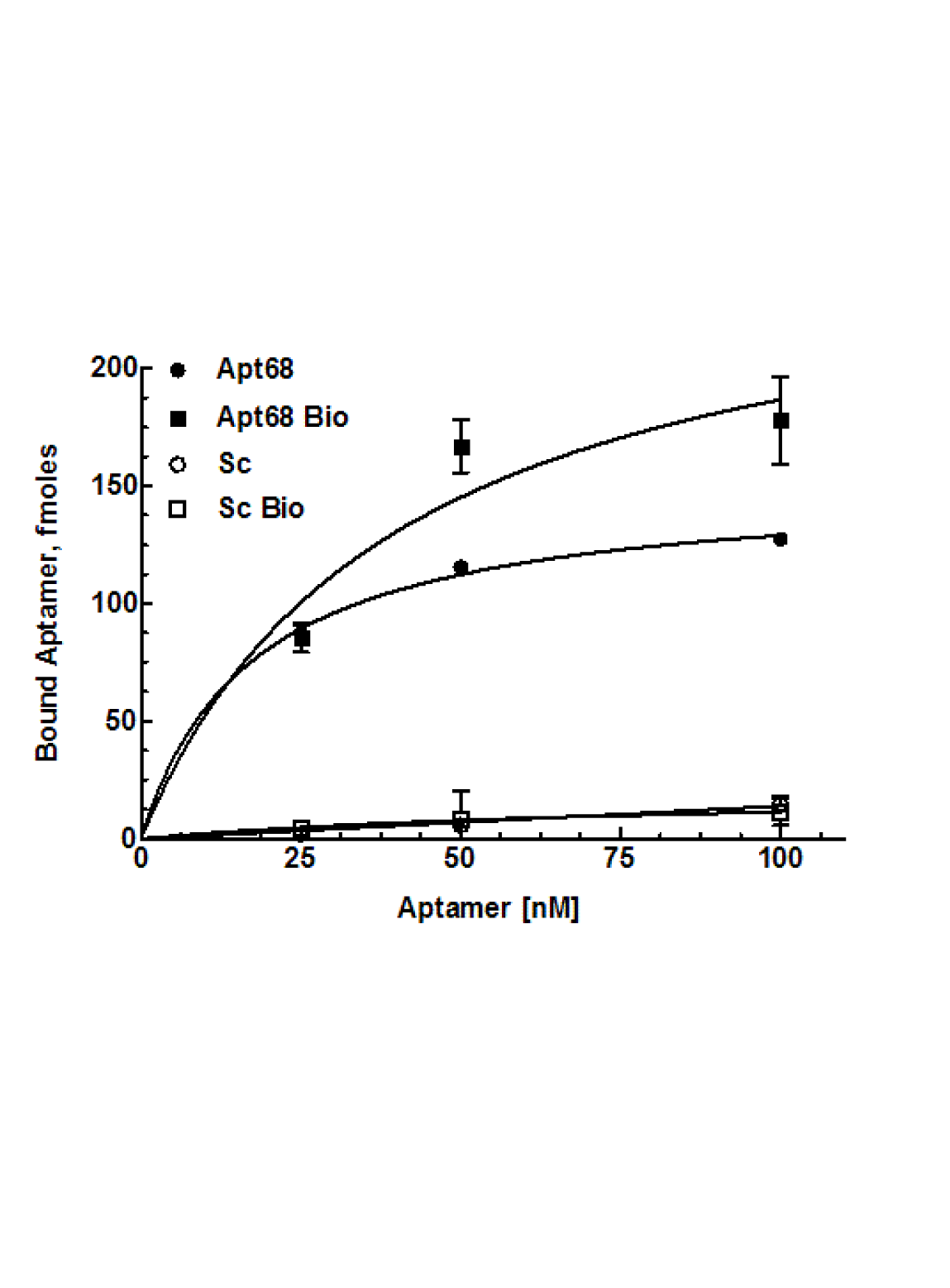

Supplement: Figure S3 — Comparison of T. cruzi trypomastigote binding activity of Apt68 and Biotinylated Apt68. 32P-GTP labeled Apt68 with and without incorporated biotin-11-ATP was used in a dose dependent binding assay with trypomastigotes. 32P-GTP labeled scrambled Apt68 with and without incorporated biotin-11-APT were used as corresponding controls. The scrambled aptamers did not show significant binding to T. cruzi trypomastigotes while both the biotinylated and non-biotinylated Apt68 bound in a dose dependent manner with high affinity. Each data point represents duplicate values and the error bars represent the standard deviation. (TIF) [file pone.0043533.s003.tif]
