# Supplementary material for: Development of an Aptamer-Based Concentration Method for the Detection of Trypanosoma cruzi in Blood
Source: PLoS One. 2012 Aug 22;7(8):e43533. doi: 10.1371/journal.pone.0043533 (PMC3425475; doi:10.1371/journal.pone.0043533)
Supplement: Table S1 — Parameters used for the T. cruzi trypomastigote whole cell SELEX. For the first round of SELEX, trypomastigotes at 1×10^8^/ml were incubated with 80 pmoles of the random RNA pool for 1 hour on ice. The trypomastigotes were recovered by centrifugation and RNA isolated from the pellet. In subsequent rounds, conditions for binding and dissociation were gradually increased in stringency by incorporating 5% FBS and salmon sperm DNA as inhibitors of non-specific interactions. As a means of negative selection and to remove aptamers that bound to targets of host origin, from round 4 of the SELEX, the aptamer pool was pre-incubated with human whole blood (RBC, platelets, leucocytes and plasma) and fetal bovine serum. After incubation for 30 minutes, this suspension was centrifuged and the supernatant incubated with T. cruzi trypomastigotes. Additionally, the binding time for the aptamers to trypomastigotes was reduced, while the number of washes and the total volume of SELEX buffer used for the washes was increased. These changes in the binding and washing protocols were made so that those aptamers in the library, that had a high association rate and a low dissociation rate, were selected. Together, these changes would result in the selection of aptamers with high affinities. The SELEX was performed for a total of 12 rounds as shown in the table. (DOC) [file pone.0043533.s004.doc]

| **SELEX Rounds** | ***T. cruzi* parasites** | **Aptamer Pool (pmoles)** | **Non-specific**  **competitor** | **Binding time** | **Wash Conditions [time per wash, number of washes, total volume of wash]** |
| --- | --- | --- | --- | --- | --- |
| R1 | 1x10^8^ | 80 | None | 1 hr | None |
| R2 | 1x10^8^ | 80 | None | 1 hr | 5min, 1x, 1ml |
| R3 | 1x10^7^ | 80 | None | 1 hr | 5min, 1x, 1ml |
| R4 | 2x10^6^ | 80 | 60x10^6^ RBC+ 200ng/ml DNA + 5% FBS | 30min | 5min, 3x, 3ml |
| R5 | 4x10^4^ | 80 | 60x10^6^ RBC+ IMDM Only | 30min | 5min, 3x, 3ml |
| R6 | 4x10^4^ | 80 | 60x10^6^ RBC+ 5% FBS | 30min | 5min, 3x, 3ml |
| R7 | 8x10^4^ | 40 | 60x10^6^ RBC+ 200ng/ml DNA + 5% FBS | 45min | 5min, 3x, 3ml |
| R8 | 10x10^4^ | 40 | 50x10^6^ RBC+ 200ng/ml DNA + 5% FBS | 30min | 30min, 1x, 10ml ; 60min, 1x, 10ml |
| R9 | 2x10^4^ | 40 | 200ng/ml DNA + 5% FBS | 15min | 5min, 1x, 10ml; 30min, 2x, 20ml, 45min, 3x, 30ml |
| R10 | 2x10^4^ | 40 | 200ng/ml DNA + 5% FBS | 5min | 30min, 5x, 50ml |
| R11 | 2x10^4^ | 40 | 200ng/ml DNA + 5% FBS | 5min | 30min, 5x, 50ml |
| R12 | 2x10^4^ | 40 | 200ng/ml DNA + 5% FBS | 5min | 30min, 6x, 60ml |

**Table S1**
